# Bi-allelic inactivation is more prevalent at relapse in multiple myeloma, identifying *RB1* as an independent prognostic marker

**DOI:** 10.1038/bcj.2017.12

**Published:** 2017-02-24

**Authors:** S S Chavan, J He, R Tytarenko, S Deshpande, P Patel, M Bailey, C K Stein, O Stephens, N Weinhold, N Petty, D Steward, L Rasche, M Bauer, C Ashby, E Peterson, S Ali, J Ross, V A Miller, P Stephens, S Thanendrarajan, C Schinke, M Zangari, F van Rhee, B Barlogie, T I Mughal, F E Davies, G J Morgan, B A Walker

**Affiliations:** 1 The Myeloma Institute, University of Arkansas for Medical Sciences, Little Rock, AR, USA; 2 Foundation Medicine Inc., Cambridge, MA, USA; 3 Albany Medical College, Albany, NY, USA; 4 Icahn School of Medicine at Mt. Sinai, New York, NY, USA; 5 Tufts University Medical Center, Boston, MA, USA

## Abstract

The purpose of this study is to identify prognostic markers and treatment targets using a clinically certified sequencing panel in multiple myeloma. We performed targeted sequencing of 578 individuals with plasma cell neoplasms using the FoundationOne Heme panel and identified clinically relevant abnormalities and novel prognostic markers. Mutational burden was associated with maf and proliferation gene expression groups, and a high-mutational burden was associated with a poor prognosis. We identified homozygous deletions that were present in multiple myeloma within key genes, including *CDKN2C*, *RB1, TRAF3, BIRC3* and *TP53*, and that bi-allelic inactivation was significantly enriched at relapse. Alterations in *CDKN2C, TP53, RB1* and the t(4;14) were associated with poor prognosis. Alterations in *RB1* were predominantly homozygous deletions and were associated with relapse and a poor prognosis which was independent of other genetic markers, including t(4;14), after multivariate analysis. Bi-allelic inactivation of key tumor suppressor genes in myeloma was enriched at relapse, especially in *RB1*, *CDKN2C* and *TP53* where they have prognostic significance.

## Introduction

Segmenting multiple myeloma (MM) into subgroups with a distinct pathogenesis and clinical behavior is an important challenge in efforts to improve clinical outcomes by optimal integration of targeted therapy. Five major translocation groups have been identified, which have a varying effect on prognosis: t(4;14), t(6;14), t(11;14), t(14;16) and t(14;20).^1^ Translocations into the *IGH* locus are primary events, present in every cell, and are present in 40–50% of myeloma patients.^2^ Secondary translocations involving the *MYC* locus are present in up to 20% of newly diagnosed patients and have a significant negative impact on prognosis.^3, 4, 5, 6, 7, 8^ Those patients without a primary translocation are generally hyperdiploid with trisomies of odd-numbered chromosomes. In addition, there are several key copy number abnormalities that have an effect on prognosis, including deletion of *CDKN2C* (1p32.3) and *TP53* (17p13.1) as well as gain or amplification of 1q21.^9, 10, 11, 12, 13^ Monosomy of chromosome 13, present in up to 50% of patients, was initially identified as a poor prognostic marker,^14^ but this was later shown to be due to its association with the t(4;14).^11, 15^

Gene expression profiling (GEP) of CD138^+^ plasma cells has further described the molecular heterogeneity in MM and can reliably subdivide MM into distinct molecular subtypes.^8, 16, 17^ Using this technology it is also possible to risk stratify MM and identify 15% of presenting cases with a very poor prognosis. By integrating both gene expression and DNA mapping arrays, homozygous deletions and an expression signature associated with apoptosis have been identified.^9, 12, 18^

Mutations have also been identified as prognostic markers on a total of ~700 cases.^4, 19, 20, 21^ The most frequently mutated genes are *NRAS* (29%), *KRAS* (23%) and *BRAF* (7%), implicating the RAS pathway as a major driver in MM. Mutations affecting prognosis have been identified, including *TP53*, *CCND1*, *ZFHX4* and *ATM*/*ATR*, but these affect small numbers of patients.^4^

The use of comprehensive gene profiling in clinical oncology has increased in recent years for the diagnosis, prognosis and prediction of response to targeted therapies. Although research institutes may design and implement their own custom panels this can be costly and labor intensive, limiting their use to large centers. In order for patients at all centers to benefit from targeted sequencing of tumors several commercial panels are available. One such effort is FoundationOne Heme (F1H), which comprises 405 genes for the analysis of single nucleotide variants, indels, copy number changes and rearrangements.^22^

To date, myeloma sequencing studies have not reported the spectrum of homozygous deletions, which clinical panels are better optimized to detecting in key pathological genes. However, we recently analyzed a set of 33 MM patients enrolled in total therapies and showed an enrichment of bi-allelic inactivation of tumor suppressor genes in high-risk cases and at relapse.^23^ In this study we have used the commercially available F1H panel^22, 24^ to determine the mutational spectra of known oncogenes and tumor suppressor genes in 578 cases consisting of monoclonal gammopathy of undetermined significance (MGUS), smoldering MM (SMM) and MM, and investigated their association with disease risk status, molecular subgroup and disease stage.

## Materials and Methods

### Patient samples and nucleic acid extraction

We report on 578 samples from individuals diagnosed with MGUS (*n*=19), SMM (*n*=42) or MM (*n*=517; 87 newly diagnosed (NDMM), 107 after treatment (TRMM) and 323 at relapse (RLMM)) who underwent targeted sequencing with the F1H assay^22^ between September 2013 and June 2015. All patients signed a written informed consent in keeping with institutional, federal and Helsinki Declaration guidelines as approved by the University of Arkansas for Medical Sciences institutional review board. Tumor samples were obtained from bone marrow aspirates, enriched by CD138^+^ selection using magnetic beads (AutoMACs, Miltenyi Biotech, Cologne, Germany or RoboSep, StemCell Technologies, Vancouver, Canada). RNA and DNA were extracted using the AllPrep DNA/RNA mini kit (Qiagen, Hilden, Germany), RNeasy RNA extraction kit (Qiagen) or Puregene DNA extraction kit (Qiagen).

### F1H reporting

Extracted DNA of ⩾50 ng or RNA of >300 ng was processed on the F1H Panel (Foundation Medicine, MA, USA). The current panel analyzes the complete coding DNA sequence of 405 genes, as well as selected introns of 31 genes involved in chromosomal rearrangements. It also interrogates the RNA sequence of 265 commonly rearranged genes resulting in gene fusions. Genes included in this assay encode known or likely targets of therapy, either FDA-approved or in clinical trials, or are otherwise known drivers of oncogenesis.

Sequencing was to an average depth of 468 × (range: 29–3781) and was performed using the Illumina HiSeq 2500. Further methods for variant calling and determination of tumor burden are detailed in the Supplementary Methods.

Sequences were analyzed for base substitutions, indels, copy number alterations (focal amplifications with ⩾8 copies and homozygous deletions) and selected gene rearrangements. Variant processing is described elsewhere^22^ but importantly involved removal of germline variants from the 1000 Genomes Project (dbSNP135), as a matched patient non-tumor sample is not used to identify truly somatic variants. All inactivating events (that is, truncations and deletions) in known tumor suppressor genes were also called as significant. To maximize mutation-detection accuracy (sensitivity and specificity) in clinical specimens, the test has been optimized and validated to detect base substitutions at a ⩾5% variant allele frequency (VAF) and indels with a ⩾10% VAF to ⩾99% accuracy. However, mutations are reported down to 1% VAF where the variant is a known hotspot and there is sufficient purity and sequencing depth. Reports were generated by Foundation Medicine and in addition data files containing additional information (VAF, variant type, depth at variant location, genomic coordinates) were received.

Comparisons with previous data sets are shown in Supplementary Results and Supplementary Table 1, and Supplementary Figure 1.

### Gene expression profiling

GEP using Affymetrix U133 Plus 2.0 arrays was performed on 503 (448 MM, 38 SMM, 17 MGUS) out of the 578 patients. The GEP based 70 risk score (GEP70), and molecular subgroups were determined.^17, 25^

### Statistical methods

Fisher's exact test of independence was used to identify significant associations of proportions of mutations and molecular subgroups, risk subgroups or molecular pathways. Multiple testing correction was performed using the false discovery rate method. The association between a mutated gene and molecular subgroups, risk subgroups or molecular pathway was considered significant if the false discovery rate adjusted *P*-value was >5%. Genes that constituted the DNA repair pathway, NF-κB pathway, MAPK pathway, epigenetic modifiers and IMiD response genes are listed in Supplementary Tables 2–6.

The log-rank test and Cox regression models were used to investigate the impact of mutations in specific genes on overall survival. Stepwise multivariate Cox regression analysis was performed using all the F1H panel genes and significant associations were graphically represented using Kaplan–Meier curves.

The Wilcoxon test was used to determine whether or not the difference between counts of alterations between two or more patient groups was significant.

Statistical analyses for Fisher's, Cox regression, Wilcoxon test and log-rank test were carried out using the R software package 3.1.3. In all statistical tests, an effect was considered statistically significant if the *P*-value for its corresponding statistical test was >5%.

## Results

### Mutational burden is associated with poor outcome

**Figure 1:**
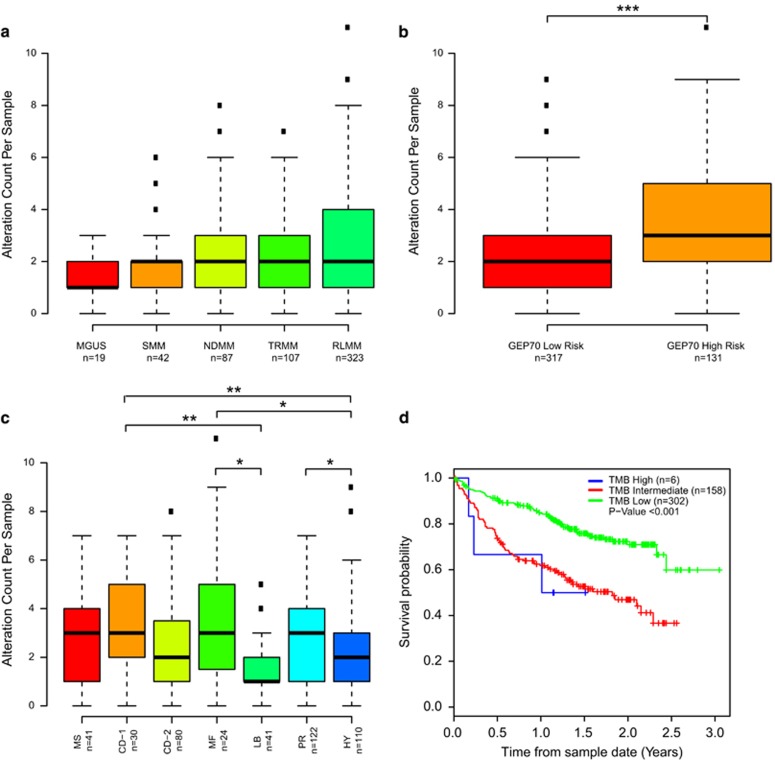
Mutational load across disease stage and gene expression groups. (**a**) Mutational count by disease stage. (**b**) Mutational count split by GEP70-defined risk group. (**c**) Mutational count split by UAMS molecular subgroups. (**d**) Mutational burden affects prognosis. *P<0.05, **P<0.01, ***P<0.001.

In 578 patient samples, we identified a total of 1381 alterations in 223 genes with an average of 3 gene alterations per sample (range 1–9) at VAFs ranging from 0.01 to 0.99. When split by disease time point there was an increase in the median number of mutations as the disease progressed, with more at relapse than at the MGUS or SMM stage (Figure 1a). We have previously performed GEP on the majority of patients with symptomatic myeloma (448/517) and analyzed these samples using the GEP70 signature^25^ and molecular subgroup classifications.^17^ There was a significant difference in the number of mutations by risk status, with more identified in GEP70 high risk than low-risk patients (*P*⩽0.001; Figure 1b). As previously seen with whole exome sequencing,^5^ there was a higher mutational load in the maf molecular subgroup (Figure 1c), which is related to the t(14;16) and increased APOBEC expression, compared to the LB and HY groups (*P*=0.03 and 0.02, respectively). The difference in mutational load was not as great as in whole exome sequencing due to the limited gene set in this targeted panel. There were also significantly more mutations in CD-1 versus LB (*P*<0.01), CD-1 versus HY (*P*<0.01) and PR versus HY (*P*<0.05) groups. Tumor mutational burden was calculated and there was a clear association with tumor burden and survival, where intermediate and high burden resulted in a worse overall survival (Figure 1d). Microsatellite instability data were available for 69 samples (Supplementary Methods), but there was no indication of microsatellite instability.

### Associations of alterations with risk groups

**Figure 2:**
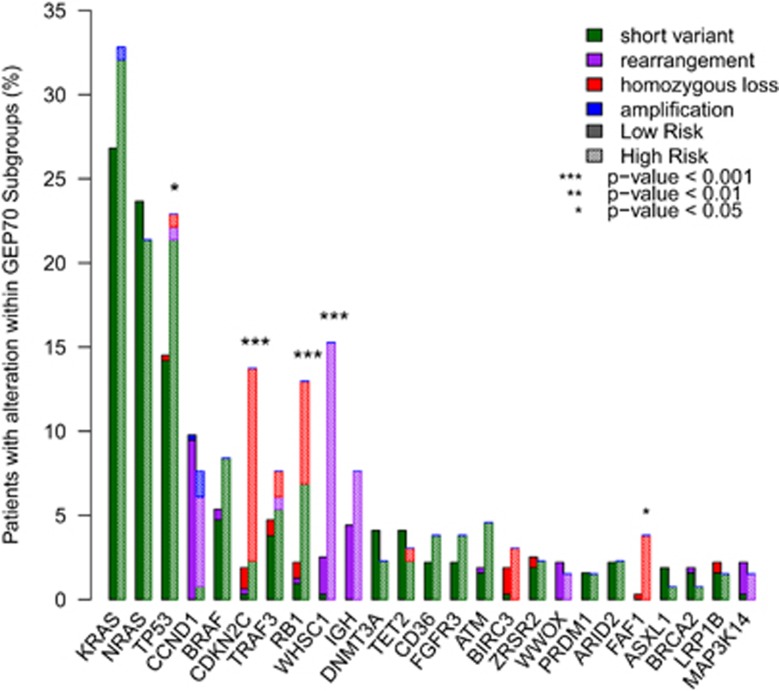
Frequency of altered genes. Frequency of altered genes separated by GEP70-risk group. Alterations are split by type. Solid bars indicate low risk and hatched bars indicate high risk.

The spectrum of mutations was similar to previous findings^4, 19, 20, 21^ and the 10 most frequently altered genes were *KRAS* (28.8%), *NRAS* (23.2%), *TP53* (17.4%), *BRAF* (6.8%), *CDKN2C* (6.0%), *RB1* (5.8%), *TRAF3* (5.8%), *DNMT3A* (3.9%), *TET2* (3.7%) and *ATM* (2.5% Figure 2 and Supplementary Table 7). *FAF1* was also frequently deleted and these samples were a subset of those with *CDKN2C* deletion, which has previously been described.^12^ Of these genes, GEP70-defined high-risk samples had a significantly higher frequency of alterations in *TP53*, *CDKN2C*, *RB1*, *WHSC1* and *FAF1* compared to low-risk samples (Figure 2).

We identified correlations between gene alterations and GEP-defined subgroups (Table 1). As expected there were correlations with alterations in translocation partner oncogenes and the GEP subgroups they define, for example, *FGFR3* and *WHSC1* alterations and the MS subgroup due to the t(4;14) rearrangement. High risk, as defined by GEP70, was associated with t(4;14), homozygous loss of *CDKN2C* and homozygous loss or mutation of *RB1*. Importantly, we described an association between *RB1* and *CDKN2C* alterations and the PR (proliferation) subgroup. Alterations in these cell cycle control genes were mainly homozygous losses that would result in progression through the G1/S phase and result in increased proliferation. There was also a correlation between alterations in *SF3B1*, which is frequently mutated in myelodysplastic syndromes, and the CD-2 subgroup. High tumor burden was correlated with the maf subgroup and intermediate tumor burden with the PR subgroup.

### Bi-allelic inactivation are associated with relapse

In addition to translocations and mutations, structural gains and losses are strong prognostic indicators in myeloma. In total, homozygous losses were detected in 72 samples with the frequency increasing as the disease progresses (MGUS *n*=0 (0%), SMM *n*=4 (9.5%), NDMM *n*=9 (10.3%), RLMM *n*=52 (16.1%)). The most frequent homozygous deletions were in *CDKN2C/FAF1*, *RB1*, *BIRC3*, *TRAF3* and *TP53*. Homozygous loss of *CDKN2C* and/or *FAF1* was detected in 26 (5.0%) samples, of which 19 were at relapse. *CDKN2C* was mutated in another four samples, of which two had VAF>0.74 indicative of bi-allelic inactivation and were also at relapse (4.6% NDMM, 6.5% RLMM). Homozygous loss of *CDKN2A/CDKN2B* was seen in another three patients, all at relapse, with mutations in six additional patients, all of which had VAFs <0.5 indicating monoallelic inactivation.

At 17p, we identified homozygous loss in 2 samples, both RLMM, with mutations in another 88 samples. Of the 88 samples with mutations, 19 had mutations with VAF>0.58 and 15/22 were in RLMM samples, indicating bi-allelic inactivation of *TP53* is a marker of relapse (0% NDMM, 5.9% RLMM). Overall, alterations in *TP53* are present in 9.1% of NDMM rising to 21.3% at relapse.

Homozygous loss of *RB1* was detected in 15 MM samples (10/15 in RLMM) with mutation in another 14 samples (10/14 in RLMM). In 11 of the samples with mutation the VAF was >0.57 (range 0.57–0.98) indicating hemizygous loss and bi-allelic inactivation (4.6% NDMM, 5.6% RLMM).

**Figure 3:**
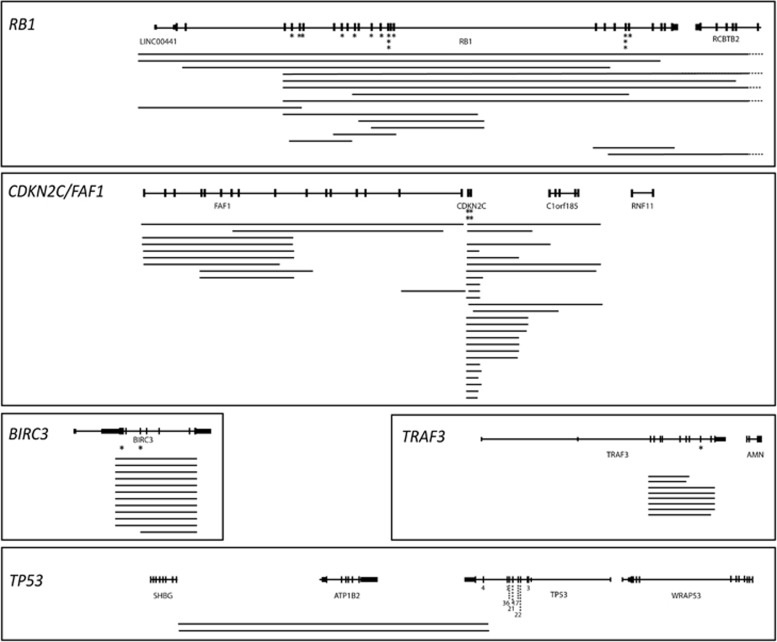
Homozygous deletion maps of *RB1*, *CDKN2C/FAF1*, *TRAF3, TP53* and *BIRC3*. Each line represents a homozygous deletion and lines on the same level are from the same patient. Dashed line indicates the deletion extends off the map. Asterisks indicate short variants, except for *TP53* where the number of variants in each exon is indicated for clarity. Data shown includes all stages of disease.

Other homozygous losses frequently detected were in *TRAF3* (8/578; 5/8 RLMM) and *BIRC3* (9/578; 8/9 RLMM) (Figure 2). *TRAF3* was mutated in an additional 27 samples, 7 of which had a VAF>0.58 (4/7 RLMM) and *BIRC3* was mutated in 2 additional samples (1/2 RLMM) both with VAF<0.5. Maps of homozygous deletions are shown in Figure 3 and mutations in common genes in Supplementary Figure 2.

Examining bi-allelic events in *CDKN2C*, *TP53* and *RB1,* by both homozygous deletion and monosomy with accompanying mutation, the rate of bi-allelic inactivation increases from 9.2% in NDMM to 17.9% at RLMM (*Z*-score *P*=0.049).

### Detection of prognostically relevant alterations using a clinical sequencing panel

**Figure 4:**
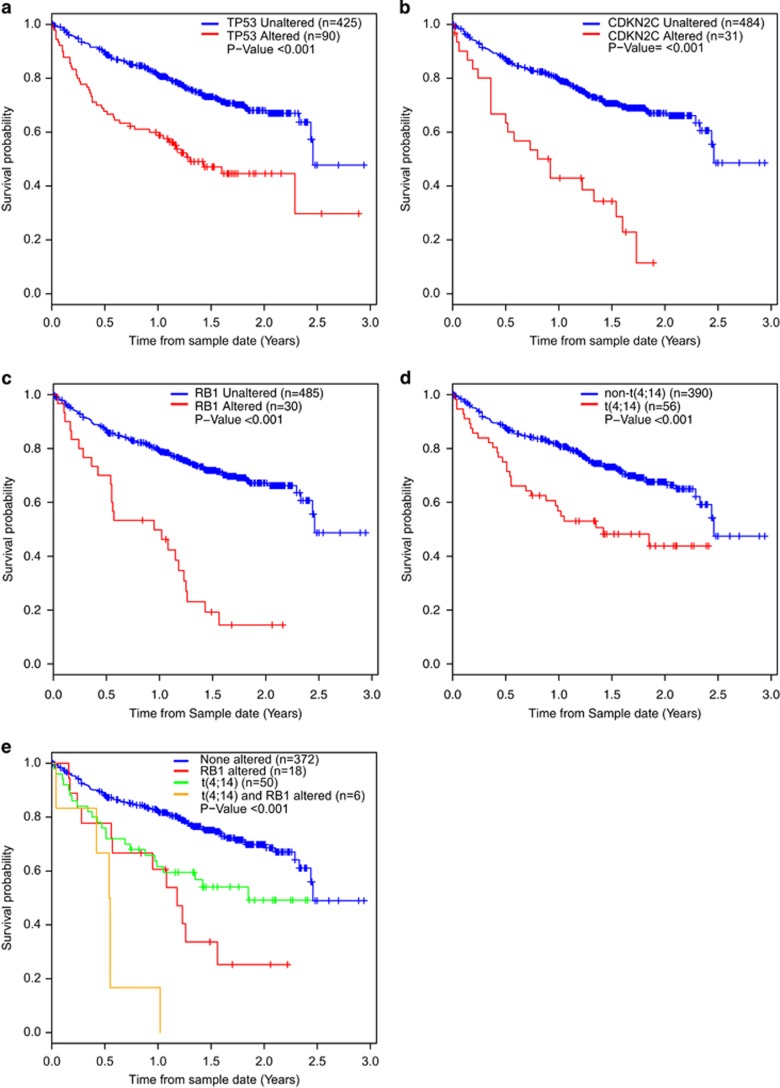
Kaplan–Meier curves for overall survival for alterations significant in univariate and multivariate analysis. (**a**), *TP53*; (**b**), *CDKN2C*; (**c**), *RB1*; (**d**), t(4;14); (**e**), combination of t(4;14) and homozygous loss of *RB1*.

To define the role of the panel in the clinic we examined the impact of genomic data on outcome and to enhance this we incorporated GEP data to define translocations. In a univariate Cox regression analysis carried out for each gene with at least six patients having an alteration, we identified *TP53*, *CDKN2C/FAF1* and *RB1* plus the t(4;14) associated with a significantly worse outcome (Figures 4a–e and Table 2). Of these, *FAF1* and *CDKN2C* were combined as patients with a *FAF1* loss also had loss of *CDKN2C*. This showed that the t(4;14) (both by GEP and sequencing rearrangement), and mutations/loss of *CDKN2C/FAF1*, *RB1* and *TP53* are associated with a significantly inferior prognosis. After multivariate Cox regression analysis the t(4;14), mutation/loss of *TP53*, *CDKN2C/FAF1* and *RB1* remain significant (Table 2). When the MM samples are split according to type (NDMM, TRMM, RLMM) the effect on survival of each alteration is more pronounced at relapse, but still present at diagnosis for *CDKN2C* and t(4;14) (Supplementary Figures 2–5). The effect of *RB1* and *TP53* alteration on survival is lost at diagnosis because the numbers of patients were small (*n*=5 and *n*=8, respectively). *CDKN2C/FAF1* and *TP53* are known prognostic markers but the prognostic significance of *RB1* has not been defined previously. Previous data has shown that almost all cases with a t(4;14) have monosomy of chromosome 13 leading to loss of *RB1* as a prognostic marker in multivariate analyses.^11, 12, 14, 15^ Here we detect homozygous deletion or mutation of *RB1*, which was associated with a poor prognosis as well as with the PR subgroup. To confirm that the prognostic effect of *RB1* is not due to association with t(4;14) we split the samples based on presence/absence of each alteration (Figure 4e) and show that patients with either the t(4;14) or alteration of *RB1* were associated with a poor prognosis, which was worse when both lesions were present.

### Identification of therapeutic targets in myeloma patients

The F1H assay is aimed at identifying important genetic abnormalities for which targeted treatments are available. We compared our data set to a list of therapies with genomic targets in any cancer and found 331 patients (64.0%) with potentially targetable alterations encompassing 38 genes. The genes and their therapies are listed in Supplementary Table 8. Most of these involved alterations of *KRAS*, *NRAS* or *BRAF* (*n*=273).

## Discussion

Here we show that the F1H assay can be used to direct patients treatment and identify clinically relevant markers. We confirm an important role for bi-allelic inactivation of key genes in myeloma at relapse, including *CDKN2C*, *TP53*, *RB1, TRAF3* and *BIRC3*. Homozygous deletion of these genes has previously been identified through the use of mapping arrays.^9, 12, 18, 26, 27^ and *CDKN2C* and *TP53* are well-accepted poor prognostic markers in myeloma.^9, 12, 28, 29^ The identification of *RB1* as a prognostic marker is more controversial as the association of monosomy of *RB1* with poor outcome has fluctuated in recent years. When monosomy of 13q was first identified it was a poor prognostic marker, but upon further analysis with other lesions it became clear that the association with poor prognosis was due to co-segregation with del(17p) and t(4;14).^11, 12, 14, 15^ Where we also show that the poor prognostic effect of *RB1* is driven by bi-allelic inactivation. Bi-allelic inactivation of *RB1* as a prognostic factor has not been described in myeloma before potentially for two reasons: homozygous deletion rates are low and the additional information provided by the identification of bi-allelic inactivation, through deletion and mutation, adds significantly to the prognostic information. *RB1* seems to be the key target for homozygous deletion on 13q as *DIS3*, also located on 13q, is frequently mutated but no homozygous deletion events were detected.

Bi-allelic inactivation was also seen in *CDKN2C* and *TP53*, and taken together with *RB1* we show a significant increase in bi-allelic inactivation in these genes from NDMM to RLMM. This indicates that bi-allelic inactivation is a key mechanism in disease progression. Bi-allelic inactivation of genes is common in cancer, including *ATM* in chronic lymphocytic leukemia,^30^ CDKN2A and CDKN2B in glioblastoma,^31^ and are indicative of loss of function of key tumor suppressor genes.^32^

Inactivation of *CDKN2C* and *RB1* are associated with the PR subgroup, which are characterized by a high proliferation index. Both of these genes are involved in cell cycle regulation, where inactivation would result in progression through G1/S phase and increased proliferation. Further investigation of *CDKN2C* and *RB1* mutation, deletion and expression are required to more fully understand the interplay between disruption of these genes and cell cycle control in myeloma.

The ability to identify bi-allelic inactivation is one of the major strengths of the F1H technology and importantly for the determination of high-risk behavior the association of bi-allelic inactivation of *CDKN2C/FAF1*, *TP53* and *RB1* with GEP70 is striking. Given that this is a targeted panel there are no data on other potentially important homozygous deletions in myeloma, such as *FAM46C* and *CYLD* both of which have been shown to be biologically or clinically important.^10, 12, 26, 27^

We have previously identified mutations or deletion of *TP53*, mutations in *ATM/ATR* and *CCND1* as well as *MYC* translocations as adversely affecting overall survival.^4^ In this data set we did not find mutation in *CCND1* or *ATM/ATR* to have a prognostic significance. This may be due to the way in which variants are called on the F1H assay, where only clinically relevant, well-characterized variants are annotated. Variants of unknown significance were not analyzed where they are not clinically relevant or where it is difficult to determine if the mutations are somatic. We confirmed the role of the poor prognostic markers t(4;14), and alterations in *CDKN2C*, and *TP53*. We have previously shown that the poor prognostic effect of the t(4;14) is somewhat negated by the use of bortezomib,^8^ but this cohort of patients were not uniformly treated and were not all part of the Total Therapy trials.

In conclusion, we have shown that bi-allelic inactivation is more prevalent at relapse in multiple myeloma and that homozygous loss of *RB1* is an independent prognostic marker.

## Figures and Tables

**Table 1:** Association of gene alterations with gene expression classifiers

*Marker*	*Classifier*	*GEP subgroup*	*Correlation*	*Adjusted* P*-value*
*CCND1*^a^	UAMS	CD-1	0.38	<0.001
*CCND1*^a^	UAMS	CD-2	0.28	<0.001
*CDKN2C*^b^	UAMS	PR	0.19	0.033
*SF3B1*^c^	UAMS	CD-2	0.23	0.029
*WHSC1*^a^	UAMS	MS	0.54	<0.001
*WWOX*^a^	UAMS	MF	0.60	<0.001
*CDKN2C*^b^	GEP70	High risk	0.24	<0.001
*RB1*^b^^,^^c^	GEP70	High risk	0.22	0.0012
*WHSC1*	GEP70	High risk	0.25	<0.001
*CCND1*^a^	TC	t(11;14)	0.30	<0.001
*CCND1*^a^	TC	t(11;14)	0.43	<0.001
*FGFR3*	TC	t(4;14)	0.44	<0.001
*IGH*^a^	TC	t(14;20)	0.30	0.02
*WHSC1*	TC	t(4;14)	0.67	<0.001
*WWOX*	TC	t(14;16)	0.77	<0.001
TMB high	UAMS	MF	0.23	0.003
TMB intermediate	UAMS	PR	0.12	0.016

Abbreviations: MF, maf; PR, proliferation; TMB, tumor burden. Shown are associations with an adjusted *P*⩽0.05.

a Rearrangements.

b Loss.

c Base substitutions.

**Table 2:** Association of gene alterations with overall survival (Cox regression)

	*Gene*	*HR*	*95% CI (lower)*	*95% CI (higher)*	*Adjusted* P*-value*
Univariate	*TP53*^a^	2.48	1.77	3.49	<0.001
	*CDKN2C*^b^	3.66	2.33	5.76	<0.001
	*RB1*^a^^,^^b^	4.07	2.63	6.29	<0.001
	*WHSC1*^c^	3.38	2.06	5.54	<0.001
	*FAF1*^b^	4.72	2.40	9.28	<0.001
	*t(4;14*)^d^	2.23	1.48	3.36	<0.001
Multivariate	*TP53*^a^	2.30	1.634	3.252	<0.001
	*CDKN2C*^b^	1.30	2.317	5.799	<0.001
	*RB1*^a^^,^^b^	1.34	2.45	5.979	<0.001
	*WHSC1*^c^	1.13	1.874	5.091	<0.001

Abbbreviations: CI, confidence interval; GEP, gene expression profiling; HR, hazard ratio.

a Base substitutions.

b Loss.

c Rearrangements.

d Predicted by GEP.
