# Supplementary material for: Bi-allelic inactivation is more prevalent at relapse in multiple myeloma, identifying RB1 as an independent prognostic marker
Source: Blood Cancer J. 2017 Feb 24;7(2):e535–. doi: 10.1038/bcj.2017.12 (PMC5386330; doi:10.1038/bcj.2017.12)
Supplement: Supplementary Information [file bcj201712x1.docx]

# Title: Bi-allelic inactivation is more prevalent at relapse in multiple myeloma, identifying *RB1* as an independent prognostic marker.

**Authors:** Shweta S Chavan^1^, Jie He^2^, Ruslana Tytarenko^1^, Shayu Deshpande^1^, Purvi Patel^1^, Mark Bailey^2^, Caleb K Stein^1^, Owen Stephens^1^, Niels Weinhold^1^, Nathan Petty^1^, Doug Steward^1^, Leo Rasche^1^, Michael Bauer^1^, Cody Ashby^1^, Erich Peterson^1^, Siraj Ali^2^, Jeff Ross^2,3^, Vincent A Miller^2^, Phillip Stephens^2^, Sharmilan Thanenderajan^1^, Carolina Schinke^1^, Maurizio Zangari^1^, Frits van Rhee^1^, Bart Barlogie^1,4^, Tariq Mughal^2,5^, Faith E Davies^1^, Gareth J Morgan^1^, Brian A Walker^1^

**Affiliations:**

1- The Myeloma Institute, University of Arkansas for Medical Sciences, 4301 W Markham, Little Rock, AR, USA.

2- Foundation Medicine Inc., Cambridge, MA

3- Albany Medical College, Albany, NY, USA

4- Icahn School of Medicine at Mt. Sinai, New York, NY 10029

5- Tufts University Medical Center, Boston, MA, USA

## Supplementary Methods

### Calculation of Tumor Mutational Burden (TMB) and Microsatellite Instability (MSI).

TMB is calculated by measuring the number of somatic mutations occurring in sequenced genes and extrapolating to the genome as a whole. The method has been shown to correlate highly with genome-wide measures of TMB.^24^ Samples are defined as high (≥20 mutations/megabase), intermediate (6-19 mutations/megabase) or low (<6 mutations/megabase).

For MSI, among the 1,897 microsatellites on the panel, the 114 that maximized variability between samples were chosen for use in the algorithm. Each chosen locus was intronic and had hg19 reference repeat length of 10-20bp. This range of repeat lengths was chosen such that the microsatellites are long enough to produce a high rate of DNA polymerase slippage, while short enough such that they are well within the 49bp read length of NGS to facilitate alignment to the human reference genome.

Using the 114 loci, the repeat length in each read that spans the locus is determined. The means and variances of repeat lengths across the reads is also calculated, forming 228 data points per sample. In a large training set of data from clinical specimens, we used principal components analysis (PCA) to project the 228-dimension data onto a single dimension (the first principal component) that maximizes the data separation, producing an NGS-based “MSI score”. There was no need to extend beyond the first principal component, as it explained ~50% of the total data variance, while none of the other principal components explained more than 4% each. Ranges of the MSI score were assigned MSI-High (MSI-H), MSI-ambiguous, or microsatellite stable (MSS) by manual unsupervised clustering of specimens for which MSI status was previously assessed either via immunohistochemistry if available or approximated by the number of homopolymer indel mutations detected by our standard pipeline. MSI-Low (MSI-L) calls are not made as there was no gold-standard test set, but presumably it would significantly overlap with our MSI-ambiguous category. For samples with low coverage (<250X median), a status of MSI-unknown is assigned.

## Supplementary Results

### Comparison with whole exome sequencing samples

The F1H panel identifies single nucleotide variants, indels, rearrangements, copy number gains (≥6 copies) and homozygous losses. Here, unless specified, we group these together as alterations. The F1H report includes either well-characterized variants that have been published elsewhere or are clinically relevant, or as variants of unknown significance (VUS) that have not been adequately defined. In order to determine which set(s) of variants to include in our analysis we compared the F1H reports with whole exome sequencing (WES) performed in-house using a matched non-tumor sample from the same patient. We found that all of the well-characterized variants were truly somatic but that 45-50% of the VUS were present in the control sample, **Supplementary Table 1**. Therefore, in this manuscript we only include the well-characterized variants for analysis. Consequently, 50-55% of those VUS discarded are truly somatic.

Amplification or gain of 1q is one of the most common structural changes in myeloma, being present in up to 40% of samples. Amplification of 1q, detected as amplification of *MCL1* at 1q21.2, was found in six patients (1%). Increase in 1q copy number is under-represented due to amplifications being detected at ≥ 6 copies, whereas in myeloma it is more usual to see 3-4 copies of 1q. It is not possible to define hyperdiploidy for the same reason.

### Mutation detection by F1H is comparable to WES for most genes

To ensure accuracy of variant calling in other genes we compared the frequency of alteration in 87 NDMM to that of 463 NDMM patients from the UK MRC Myeloma XI trial^4^. We found that the overall correlation coefficient (r) was 0.85, **Supplementary Figure 1**. Most gene alteration frequencies in the F1H dataset fell within 2.5% of the UK dataset, including *NRAS*. However there were some important differences including *DIS3,* *FAM46C* and *ATM* which were under-reported in the F1H dataset. Upon examining the sample reports there were a large number of VUS for these genes, indicating that the mutations are present but Foundation Medicine is unable to determine if they are somatic. Conversely, *TP53, CCND1, WHSC1, CDKN2C and RB1* were over-represented in the F1H dataset due to the detection of mutations and structural alterations, such as homozygous losses or rearrangements. Additionally, alterations were found at a higher frequency in the NDMM F1H dataset in *KRAS* (32.9% vs. 22.5%)*, BRAF* (11.3% vs. 7.7%) and *CD36* (3.4% vs. 0.2%). For *KRAS* these included several codons not previously reported in the two largest datasets (n=666), such as L19, Q22, L23, and T58, but these mutations were documented in the COSMIC database.^38^ Regarding *BRAF*, all variants reported had previously been documented in myeloma, and for CD36 8/14 variants involve codon Y325* which corresponds to SNP rs3211938 present in dbSNP build 144.

### Differences in the frequency of alteration between disease stages

As this dataset contains samples from different disease stages we compared the frequencies of alterations at each stage. We saw an increased frequency of Ras pathway gene mutations as the disease progresses from MGUS to SMM (15.7% to 40.4%) and from SMM to NDMM (56.32%), but there was no significant difference in the frequencies between NDMM and RLMM (53.56%), **Supplementary Figure 6**. Mutations in the Ras pathway are most frequent in myeloma. Overall 281 (48.6%) patients had alteration of *NRAS*, *KRAS* or *BRAF* (**Supplementary Figure 2**). *NRAS* alterations were predominantly at known hotspots (n=119/120), with activating mutations seen at codon G12, G13 or Q61 with an average VAF of 0.26, 0.38 and 0.27 respectively (range 0.01 to 0.97, **Supplementary Figure 7**). The codons G12, G13 and Q61 were also the most frequent targets for alterations in *KRAS* (n=129/149) average VAF 0.29, 0.26 and 0.24 respectively; (range 0.01-0.92). The frequency and distribution of these mutations was in line with results from others.^4, 19-21^ In 21 of 35 (60.0%) patients with *BRAF* alterations, the hotspot mutation V600E was found with an average VAF of 0.29 (range 0.01–0.67). We found concomitant alterations in *KRAS* and *NRAS* in 14, *KRAS* and *BRAF* in 8 and *NRAS* and *BRAF* in 4 patients. Three patients had mutations in *BRAF*, *KRAS* and *NRAS*.

The frequency of *TP53* alteration in NDMM was 9.2%, **Supplementary Figure 6**. We observed a higher frequency of *TP53* alterations in RLMM (21.9% vs. 9.2%). *TP53* alterations were relatively rare in MGUS and were present with a low variant allele frequency. Most *TP53* alterations are located in the DNA binding domain of the protein and were predicted to be deleterious. 19 (3.7%) patients had more than one *TP53* alteration (range 2-4), potentially indicating bi-allelic loss of function.

*ATM* alterations are detected in 1.1% of NDMM and show a slight increase in RLMM (3.1%) as do alterations in *ATR* (NDMM 0% to RLMM 0.3%), **Supplementary Figure 6**.

### Alterations in p53 and PI(3)K/Ras signaling pathways are enriched in MM

We carried out a network analysis to infer the mutated sub-networks of interacting genes from large cancer interaction networks as defined by pan-cancer analysis (HotNet2)^29^. The gene-set evaluated by HotNet2 comprises of preselected genes derived from large scale cancer sequencing studies from The Cancer Genome Atlas. We investigated the frequency of alterations in each of these networks at different disease stages. In 151 (26.1%) patients, alterations of genes associated with p53 signaling were detected and in 302 (52.2%) patients alterations associated with PI(3)K/Ras signaling were detected, **Supplementary** **Table 9**. The p53 signaling pathway showed a higher frequency of alterations in RLMM than in NDMM (20.6% NDMM vs. 31.8% RLRR), whereas the PI(3)K/Ras signaling pathway did not (59.7% NDMM vs. 56.6% RLMM). *TP53* alterations result in a negative impact on survival, **Table 2**. An effect on survival was seen with the PI(3)K/Ras pathway, **Supplementary Figure 8,** and was driven by patients with a mutation in *KRAS* who had been previously treated, **Supplementary Figure 9**. Additionally, we performed analyses on manually curated pathways including DNA repair and NF-κB pathways along with epigenetic modifiers and IMiD response genes for an effect on survival, **Supplementary Tables 2-6**, but none were seen.

## List of Supplementary Figures:

**Supplementary Figure 1** Comparison of the frequency of alteration in NDMM samples analyzed by F1H panel and UK MRC Myeloma XI data.

**Supplementary Figure 2** Distribution of variants in TP53, NRAS, KRAS, and BRAF

**Supplementary Figure 3** KM plots for Newly Diagnosed Samples

**Supplementary Figure 4** KM plots for Newly Relapse Samples

**Supplementary Figure 5** KM plots for Newly Treated Samples

**Supplementary Figure 6** Comparison of frequency of alterations at different disease stages.

**Supplementary Figure 7** Comparison of allele frequency of gene mutations at different disease stages in KRAS, NRAS, BRAF, and TP53

**Supplementary Figure 8** KRAS, but not NRAS or BRAF, alterations result in a worse overall survival

**Supplementary Figure 9.** Effect of KRAS mutation at A. NDMM B.TRMM C.RLMM

## List of Supplementary Tables:

**Supplementary Table 1** Comparison of F1H and exome sequencing calls.

**Supplementary Table 2** Genes comprising DNA repair pathway

**Supplementary Table 3** Genes comprising NF-κB pathway

**Supplementary Table 4** Genes comprising MAPK pathway

**Supplementary Table 5** Genes comprising Epigenetic modifiers

**Supplementary Table 6** IMiD genes

**Supplementary Table 7** Genes altered on the F1H panel with their frequencies.

**Supplementary Table 8** List of genes with targetable alterations and the associated therapies

**Supplementary Table 9** Comparison of the frequencies at different disease stages to highlight specific genetic alterations in pathways in cancer as per HotNet2

## Supplementary Tables

## Supplementary Table 1. Comparison of F1H and exome sequencing calls.

| **TRF** | **Depth** | **Gene** | **Alteration type** | **Variant status** | **Chr.** | **Position** | **c.** | **p.** | **VAF** | **Germline** | **If use unknowns** | **If discard unknowns** |
| --- | --- | --- | --- | --- | --- | --- | --- | --- | --- | --- | --- | --- |
| **TRF037844** | 439 | *DNMT3A* | short variant | unknown | 2 | 25463574 | 2108T>A | L703Q | 0.08 | Yes | False pos | True |
| **TRF037844** | 483 | *NRAS* | short variant | known | 1 | 115258747 | 35G>C | G12A | 0.16 | No | True | True |
| **TRF037844** | 565 | *KDM2B* | short variant | unknown | 12 | 121880522 | 2722G>A | D908N | 0.52 | Yes | False pos | True |
| **TRF037844** | 338 | *KDM5A* | short variant | unknown | 12 | 432253 | 2270A>G | K757R | 0.5 | Yes | False pos | True |
| **TRF037844** | 273 | *MAGED1* | short variant | unknown | 23 | 51638306 | 371C>T | S124L | 0.1 | No | True | False neg |
| **TRF037844** | 461 | *TET2* | short variant | unknown | 4 | 106155177 | 78G>C | Q26H | 0.52 | Yes | False pos | True |
| **TRF037844** | 656 | *SPEN* | short variant | unknown | 1 | 16258662 | 5927A>T | K1976M | 0.19 | No | True | False neg |
| **TRF037844** | NULL | *IGH* | rearrangement | unknown | 14 | 106327017 | NULL | NULL | NULL | No | True | False neg |
| **TRF037844** | 482 | *NCOR1* | short variant | likely | 17 | 15961248 | 6140_6141insGCTGATCACACTT | I2055fs*3 | 0.15 | No | True | True |
| **TRF037844** | 773 | *CD22* | short variant | unknown | 19 | 35827127 | 601C>T | R201W | 0.16 | No | True | False neg |
| **TRF037844** | 565 | *BRD4* | short variant | unknown | 19 | 15375255 | 1172G>A | C391Y | 0.57 | Yes | False pos | True |
| **TRF037844** | 593 | *DNM2* | short variant | unknown | 19 | 10940876 | 2365C>T | P789S | 0.17 | No | True | False neg |
| **TRF065016** | 345 | *TP53* | short variant | known | 17 | 7578508 | 422G>A | C141Y | 0.22 | No | True | True |
| **TRF065016** | 527 | *SETBP1* | short variant | unknown | 18 | 42530630 | 1325C>G | T442S | 0.19 | No | True | False neg |
| **TRF065016** | 176 | *CDKN2C* | short variant | likely | 1 | 51436135 | 96_115delTGCACAAAATGGATTTGGAA | N32fs*21 | 0.84 | No | True | True |
| **TRF065016** | 427 | *POT1* | short variant | unknown | 7 | 124503560 | 390C>A | H130Q | 0.68 | No | True | False neg |
| **TRF065016** | 317 | *LRP1B* | short variant | unknown | 2 | 141083346 | 12325G>A | V4109I | 0.5 | Yes | False pos | True |
| **TRF065016** | 127 | *WDR90* | short variant | unknown | 16 | 717437 | 5095A>G | M1699V | 0.07 | Yes | False pos | True |
| **TRF065016** | 519 | *MLL2* | short variant | likely | 12 | 49432597 | 8542C>T | Q2848* | 0.3 | No | True | True |
| **TRF065016** | 350 | *PIK3C2G* | short variant | unknown | 12 | 18800921 | 4297G>A | D1433N | 0.48 | Yes | False pos | True |
| **TRF065016** | 532 | *FBXW7* | short variant | likely | 4 | 153332662 | 275_293>GTGTTTCCT | E96fs*70 | 0.07 | No | True | True |
| **TRF065016** | 331 | *LEF1* | short variant | unknown | 4 | 108969836 | 1153G>A | A385T | 0.6 | Yes | False pos | True |
| **TRF065016** | 191 | *AR* | short variant | unknown | 23 | 66766356 | 1369_1386delGGCGGCGGCGGCGGCGGC | G457_G462del | 0.25 | Yes | False pos | True |
| **TRF065016** | 438 | *CDK8* | short variant | unknown | 13 | 26959441 | 608A>G | E203G | 0.2 | No | True | False neg |
| **TRF065016** | NULL | *RPTOR* | rearrangement | unknown | 17 | 78704229 | NULL | NULL | NULL | Yes | False pos | True |
| **TRF065016** | 399 | *NFKBIA* | short variant | unknown | 14 | 35872452 | 451G>T | A151S | 0.15 | No | True | False neg |
| **TRF065016** | 329 | *SF3B1* | short variant | unknown | 2 | 198281625 | 506G>C | R169T | 0.17 | No | True | False neg |

## Supplementary Table 2: Genes comprising DNA repair pathway

| **Gene Name** | **Function** | **Gene Description** |
| --- | --- | --- |
| ***ATM*** | DNA damage detection | ATM serine/threonine kinase |
| ***ATR*** | DNA damage detection | ATR serine/threonine kinase |
| ***BLM*** | Fanconi anemia pathway | Bloom syndrome, RecQ helicase-like |
| ***BRCA1*** | Fanconi anemia pathway | breast cancer 1, early onset |
| ***BRCA2*** | Fanconi anemia pathway | breast cancer 2, early onset |
| ***BRIP1*** | Fanconi anemia pathway | BRCA1 interacting protein C-terminal helicase 1 |
| ***CHEK1*** | DNA damage detection | checkpoint kinase 1 |
| ***CHEK2*** | DNA damage detection | checkpoint kinase 2 |
| ***FANCA*** | Fanconi anemia pathway | Fanconi anemia, complementation group A |
| ***FANCC*** | Fanconi anemia pathway | Fanconi anemia, complementation group C |
| ***FANCD2*** | Fanconi anemia pathway | Fanconi anemia, complementation group D2 |
| ***FANCE*** | Fanconi anemia pathway | Fanconi anemia, complementation group E |
| ***FANCF*** | Fanconi anemia pathway | Fanconi anemia, complementation group F |
| ***FANCG*** | Fanconi anemia pathway | Fanconi anemia, complementation group G |
| ***FANCI*** | Fanconi anemia pathway | Fanconi anemia, complementation group I |
| ***FANCL*** | Fanconi anemia pathway | Fanconi anemia, complementation group L |
| ***FANCM*** | Fanconi anemia pathway | Fanconi anemia, complementation group M |
| ***NBN*** | DS break repair | nibrin |
| ***PALB2*** | Fanconi anemia pathway | partner and localizer of BRCA2 |
| ***PARP1*** | DNA damage detection | poly (ADP-ribose) polymerase 1 |
| ***PARP2*** | DNA damage detection | poly (ADP-ribose) polymerase 2 |
| ***PARP3*** | DNA damage detection | poly (ADP-ribose) polymerase family, member 3 |
| ***PRKDC*** | DS break repair | protein kinase, DNA-activated, catalytic polypeptide |
| ***RAD50*** | DS break repair | RAD50 homolog (S. cerevisiae) |
| ***RAD51C*** | Fanconi anemia pathway | RAD51 paralog C |
| ***RAD52*** | DS break repair | RAD52 homolog (S. cerevisiae) |
| ***RAD54L*** | DS break repair | RAD54-like (S. cerevisiae) |
| ***RPA1*** | Fanconi anemia | replication protein A1, 70kDa |
| ***TP53*** | DNA damage detection | tumor protein p53 |

## Supplementary Table 3: Genes comprising NF-κB pathway

| **Gene Name** | **Function** | **Gene Description** |
| --- | --- | --- |
| ***BCL10*** | canonical NF-κB activator | B-cell CLL/lymphoma 10 |
| ***BIRC3*** | canonical NF-κB activator | baculoviral IAP repeat containing 3 |
| ***CARD11*** | Proapoptotic NF-κB activator | caspase recruitment domain family, member 11 |
| ***CHUK*** | NF-κB inhibitor | conserved helix-loop-helix ubiquitous kinase |
| ***GADD45B*** | proapoptotic | growth arrest and DNA-damage-inducible, beta |
| ***MALT1*** | canonical NF-κB activator | MALT1 paracaspase |
| ***MAP3K14*** | Non canonical NF-κB activator | mitogen-activated protein kinase kinase kinase 14 |
| ***MYD88*** | canonical NF-κB activator | myeloid differentiation primary response 88 |
| ***PIM1*** | NF-κB activator | Pim-1 proto-oncogene, serine/threonine kinase |
| ***PLCG2*** | canonical NF-κB activator | phospholipase C, gamma 2 (phosphatidylinositol-specific) |
| ***SYK*** | canonical NF-κB activator | spleen tyrosine kinase |
| ***TRAF2*** | canonical NF-κB activator | TNF receptor-associated factor 2 |
| ***TRAF3*** | canonical NF-κB activator | TNF receptor-associated factor 3 |

## Supplementary Table 4: Genes comprising MAPK pathway

| **Gene Name** | **Function** | **Gene Description** |
| --- | --- | --- |
| ***BRAF*** | RAS pathway | proto-oncogene B-Raf |
| ***KRAS*** | RAS pathway | Kirsten rat sarcoma viral oncogene homolog |
| ***NF1*** | RAS pathway | Neurofibromin 1 |
| ***NRAS*** | RAS pathway | Neuroblastoma RAS Viral (V-Ras) Oncogene Homolog |

## Supplementary Table 5: Genes comprising Epigenetic modifiers

| **Gene Name** | **Function** | **Gene Description** |
| --- | --- | --- |
| ***ARID1A*** | Chromatin Structure Regulator | AT rich interactive domain 1A (SWI-like) |
| ***ARID2*** | Chromatin Structure Regulator | AT rich interactive domain 2 (ARID, RFX-like) |
| ***BRD4*** | Epigenetic reader | bromodomain containing 4 |
| ***CHD2*** | Chromatin Structure Regulator | chromodomain helicase DNA binding protein 2 |
| ***DNMT3A*** | 5mC modifier | DNA (cytosine-5-)-methyltransferase 3 alpha |
| ***DOT1L*** | Histone Methyltransferases | DOT1-like histone H3K79 methyltransferase |
| ***EP300*** | Histone Acetyltransferase | E1A binding protein p300 |
| ***HDAC1*** | Histone Deacetylase | histone deacetylase 1 |
| ***HDAC4*** | Histone Deacetylase | histone deacetylase 4 |
| ***HDAC7*** | Histone Deacetylase | histone deacetylase 7 |
| ***HIST1H1C*** | Histone 1 protein | histone cluster 1, H1c |
| ***HIST1H1D*** | Histone 1 protein | histone cluster 1, H1d |
| ***HIST1H1E*** | Histone 1 protein | histone cluster 1, H1e |
| ***HIST1H2AC*** | Histone 1 protein | histone cluster 1, H2ac |
| ***HIST1H2AG*** | Histone 1 protein | histone cluster 1, H2ag |
| ***HIST1H2AL*** | Histone 1 protein | histone cluster 1, H2al |
| ***HIST1H2AM*** | Histone 1 protein | histone cluster 1, H2am |
| ***HIST1H2BC*** | Histone 1 protein | histone cluster 1, H2bc |
| ***HIST1H2BJ*** | Histone 1 protein | histone cluster 1, H2bj |
| ***HIST1H2BK*** | Histone 1 protein | histone cluster 1, H2bk |
| ***HIST1H2BO*** | Histone 1 protein | histone cluster 1, H2bo |
| ***HIST1H3B*** | Histone 1 protein | histone cluster 1, H3b |
| ***IDH1*** | 5mC modifier | isocitrate dehydrogenase 1 (NADP+), soluble |
| ***IDH2*** | 5mC modifier | isocitrate dehydrogenase 2 (NADP+), mitochondrial |
| ***KDM2B*** | Histone Demethylase | lysine (K)-specific demethylase 2B |
| ***KDM4C*** | Histone Demethylase | lysine (K)-specific demethylase 4C |
| ***KDM5A*** | Histone Demethylase | lysine (K)-specific demethylase 5A |
| ***KDM5C*** | Histone Demethylase | lysine (K)-specific demethylase 5C |
| ***KDM6A*** | Histone Demethylase | lysine (K)-specific demethylase 6A |
| ***MLL*** | Histone Methyltransferase | lysine (K)-specific methyltransferase 2D |
| ***MLL2*** | Histone Methyltransferase | lysine (K)-specific methyltransferase 2B |
| ***MLL3*** | Histone Methyltransferase | lysine (K)-specific methyltransferase 2C |
| ***MYST3*** | Histone Acetyltransferase | K(lysine) acetyltransferase 6A |
| ***NSD1*** | Histone Methyltransferase | nuclear receptor binding SET domain protein 1 |
| ***SETD2*** | Histone Methyltransferase | SET domain containing 2 |
| ***SMARCA4*** | Chromatin Structure Regulator | SWI/SNF related, matrix associated, actin dependent regulator of chromatin, subfamily a, member 4 |
| ***TET2*** | 5mC modifier | tet methylcytosine dioxygenase 2 |
| ***WHSC1*** | Histone Methyltransferase | Wolf-Hirschhorn syndrome candidate 1 |

## Supplementary Table 6: IMiD genes

| **Gene Name** | **Gene Description** |
| --- | --- |
| ***IRF4*** | interferon regulatory factor 4 |
| ***CRBN*** | cereblon |
| ***DDB1*** | damage-specific DNA binding protein 1, 127kDa |
| ***CUL4A*** | cullin 4A |
| ***CUL4B*** | cullin 4B |
| ***IKZF1*** | IKAROS family zinc finger 1 (Ikaros) |
| ***IKZF2*** | IKAROS family zinc finger 2 (Helios) |
| ***IKZF3*** | IKAROS family zinc finger 3 (Aiolos) |

## Supplementary Table 7. Genes altered on the F1H panel with their frequencies.

| Gene | Patient (n) | Patient (%) |
| --- | --- | --- |
| *KRAS* | 149 | 28.82 |
| *NRAS* | 120 | 23.21 |
| *TP53* | 90 | 17.41 |
| *CCND1* | 43 | 8.32 |
| *BRAF* | 35 | 6.77 |
| *CDKN2C* | 31 | 6.00 |
| *TRAF3* | 30 | 5.80 |
| *RB1* | 30 | 5.80 |
| *WHSC1* | 28 | 5.42 |
| *DNMT3A* | 20 | 3.87 |
| *TET2* | 19 | 3.68 |
| *CD36* | 14 | 2.71 |
| *FGFR3* | 14 | 2.71 |
| *ATM* | 13 | 2.51 |
| *BIRC3* | 11 | 2.13 |
| *ZRSR2* | 11 | 2.13 |
| *WWOX* | 10 | 1.93 |
| *PRDM1* | 10 | 1.93 |
| *ARID2* | 10 | 1.93 |
| *FAF1* | 10 | 1.93 |
| *ASXL1* | 9 | 1.74 |
| *BRCA2* | 9 | 1.74 |
| *LRP1B* | 9 | 1.74 |
| *MAP3K14* | 9 | 1.74 |

## Supplementary Table 8: List of genes with targetable alterations and the associated therapies

| Gene | Patient count | Targeted Therapy |
| --- | --- | --- |
| *KRAS* | 145 | Idelalisib,Trametinib |
| *NRAS* | 115 | Idelalisib,Trametinib |
| *BRAF* | 34 | Dabrafenib,Regorafenib,Sorafenib,Trametinib,Vemurafenib |
| *TET2* | 19 | Azacitidine,Decitabine |
| *FGFR3* | 14 | Pazopanib,Ponatinib |
| *PTPN11* | 9 | Idelalisib,Trametinib |
| *NF1* | 8 | Everolimus,Temsirolimus,Trametinib |
| *DNMT3A* | 5 | Azacitidine,Decitabine |
| *FBXW7* | 5 | Everolimus,Temsirolimus |
| *IDH1* | 5 | Azacitidine,Decitabine |
| *STK11* | 5 | Bosutinib,Dasatinib,Everolimus,Temsirolimus |
| *CSF1R* | 4 | Sunitinib |
| *IDH2* | 3 | Azacitidine,Decitabine |
| *TSC2* | 3 | Everolimus,Temsirolimus |
| *ALK* | 2 | Ceritinib,Crizotinib |
| *ARAF* | 2 | Sorafenib |
| *BRCA2* | 2 | Olaparib,Ponatinib,Sorafenib,Sunitinib |
| *EGFR* | 2 | Afatinib,Cetuximab,Erlotinib,Gefitinib,Lapatinib,Panitumumab |
| *FLT4* | 2 | Axitinib,Pazopanib,Regorafenib,Sorafenib,Sunitinib,Vandetanib |
| *MTOR* | 2 | Everolimus,Temsirolimus |
| *PIK3CA* | 2 | Everolimus,Temsirolimus |
| *SMO* | 2 | Vismodegib |
| *SRC* | 2 | Bosutinib,Dasatinib |
| *ABL1* | 1 | Bosutinib,Dasatinib,Imatinib,Nilotinib,Ponatinib |
| *BRIP1* | 1 | Olaparib |
| *ERBB4* | 1 | Afatinib,Erlotinib,Lapatinib |
| *JAK1* | 1 | Ruxolitinib |
| *JAK2* | 1 | Ruxolitinib |
| *MAP2K1* | 1 | Trametinib |
| *MAP2K2* | 1 | Trametinib |
| *PIK3R1* | 1 | Everolimus,Temsirolimus |
| *PIK3R2* | 1 | Everolimus,Temsirolimus |
| *PTEN* | 1 | Everolimus,Idelalisib,Temsirolimus |
| *RAF1* | 1 | Regorafenib,Sorafenib,Trametinib |
| *ROS1* | 1 | Ceritinib,Crizotinib |
| *TSC1* | 1 | Everolimus,Temsirolimus |

## Supplementary Table 9: Comparison of the frequencies at different disease stages to highlight specific genetic alterations in pathways in cancer using *HotNet2* algorithm.

| Pathway | MGUS  (C)* | MGUS  (E)* | MGUS  (ALL)* | SMM  (C)* | SMM  (E)* | SMM  (ALL)* | ND  (C)* | ND  (E)* | ND  (ALL)* | RL  (C)* | RL  (E)* | RL  (ALL)* |
| --- | --- | --- | --- | --- | --- | --- | --- | --- | --- | --- | --- | --- |
| ASCOM complex | 0 | 0 | 0 | 2 | 0 | 2 | 1 | 0 | 1 | 7 | 0 | 7 |
| BAP1 complex | 1 | 0 | 1 | 1 | 0 | 1 | 0 | 0 | 0 | 10 | 0 | 10 |
| Cohesin complex | 0 | 0 | 0 | 0 | 0 | 0 | 2 | 0 | 2 | 2 | 2 | 4 |
| Core binding factors | 0 | 0 | 0 | 0 | 0 | 0 | 0 | 0 | 0 | 2 | 0 | 2 |
| MHC Class I proteins | 0 | 0 | 0 | 0 | 0 | 0 | 1 | 0 | 1 | 0 | 0 | 0 |
| NOTCH signaling | 0 | 0 | 0 | 0 | 1 | 1 | 0 | 1 | 1 | 8 | 3 | 11 |
| P53 signaling | 6 | 0 | 6 | 6 | 1 | 7 | 16 | 2 | 18 | 94 | 9 | 103 |
| PI(3)K signaling | 2 | 2 | 4 | 14 | 5 | 19 | 37 | 15 | 52 | 102 | 81 | 183 |
| RTK signaling | 0 | 0 | 0 | 1 | 0 | 1 | 0 | 0 | 0 | 0 | 0 | 0 |
| SWI/SNF complex | 1 | 0 | 1 | 0 | 0 | 0 | 0 | 1 | 1 | 9 | 6 | 15 |
| SMARCB1, SMARCA4 | 0 | 0 | 0 | 0 | 0 | 0 | 0 | 0 | 0 | 1 | 0 | 1 |
| MYD88, SPOP | 0 | 0 | 0 | 0 | 0 | 0 | 0 | 0 | 0 | 0 | 0 | 0 |
| *C = Core pathways; E = Extended pathways; ALL = Core and Extended pathways | | | | | | | | | | | | |

## Supplementary Figure 1. Comparison of the frequency of alteration in NDMM samples analyzed by F1H panel and UK MRC Myeloma XI data. Red line indicates complete correlation and black lines indicate 2.5% variance. Genes with >2.5% variance are labeled.


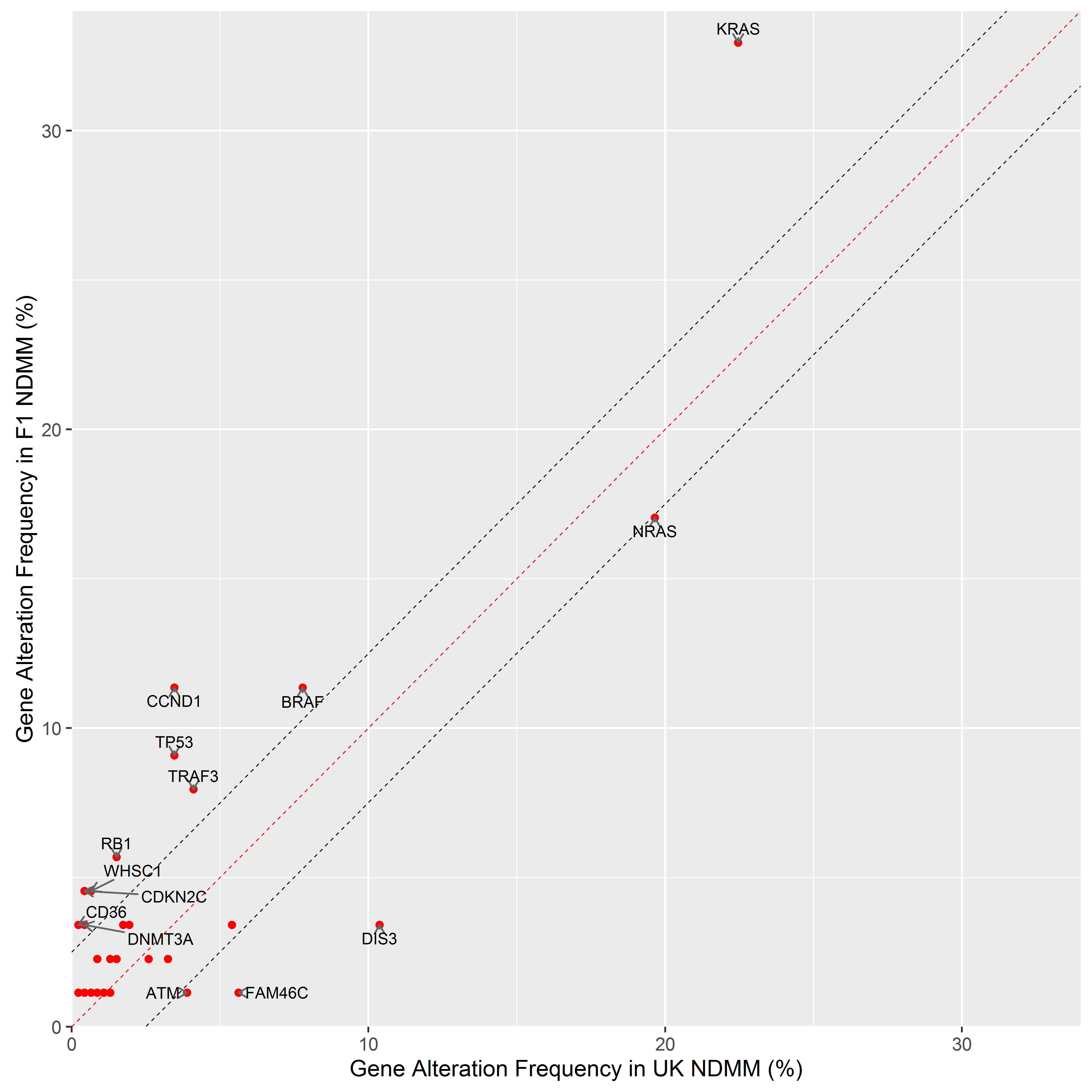


## Supplementary Figure 2. Distribution of variants in *TP53, KRAS, BRAF*, and *NRAS*.


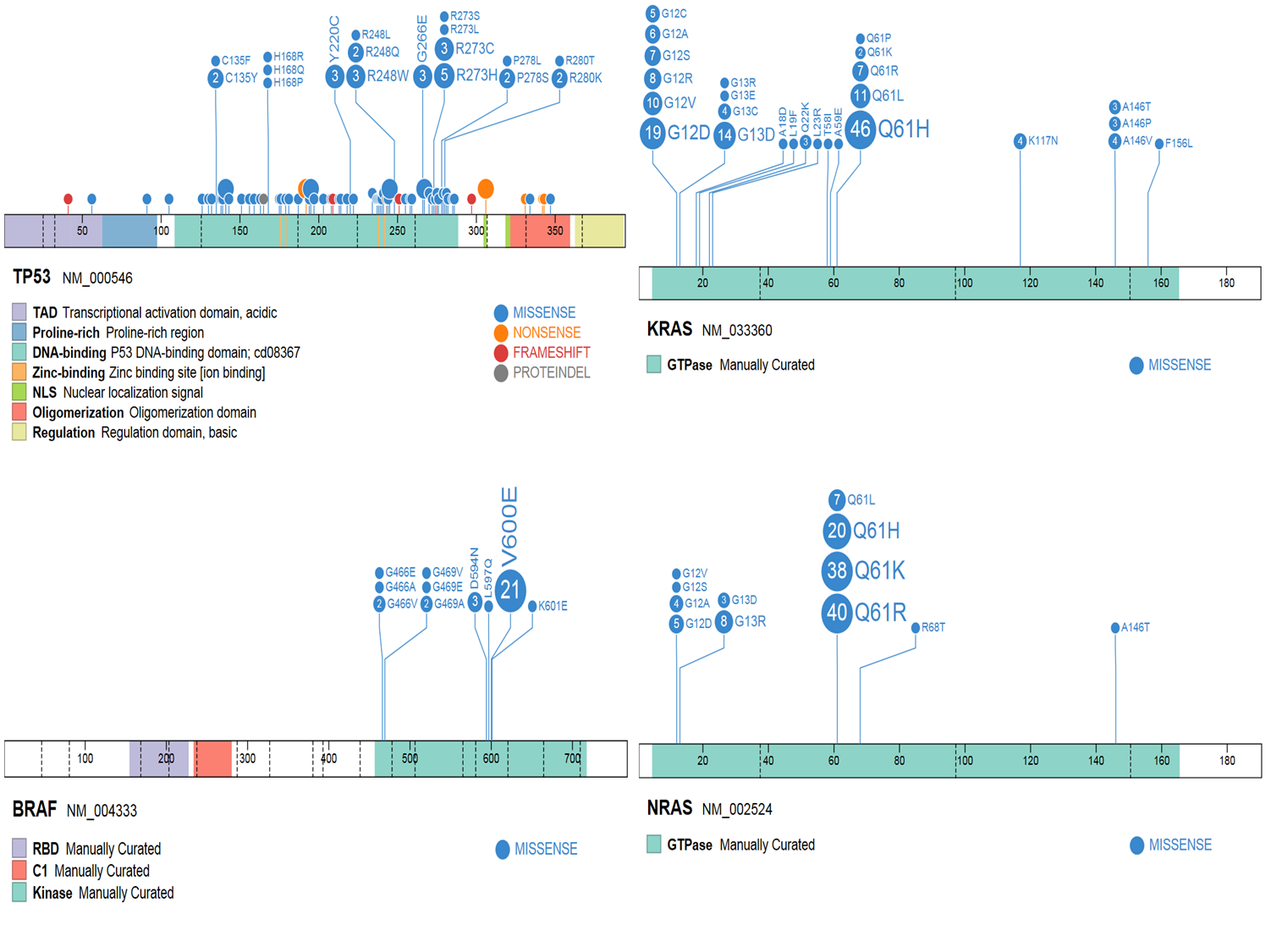


## Supplementary Figure 3. Kaplan-Meier plots for Newly Diagnosed Myeloma (NDMM) for genes significant in multi-variate analysis.


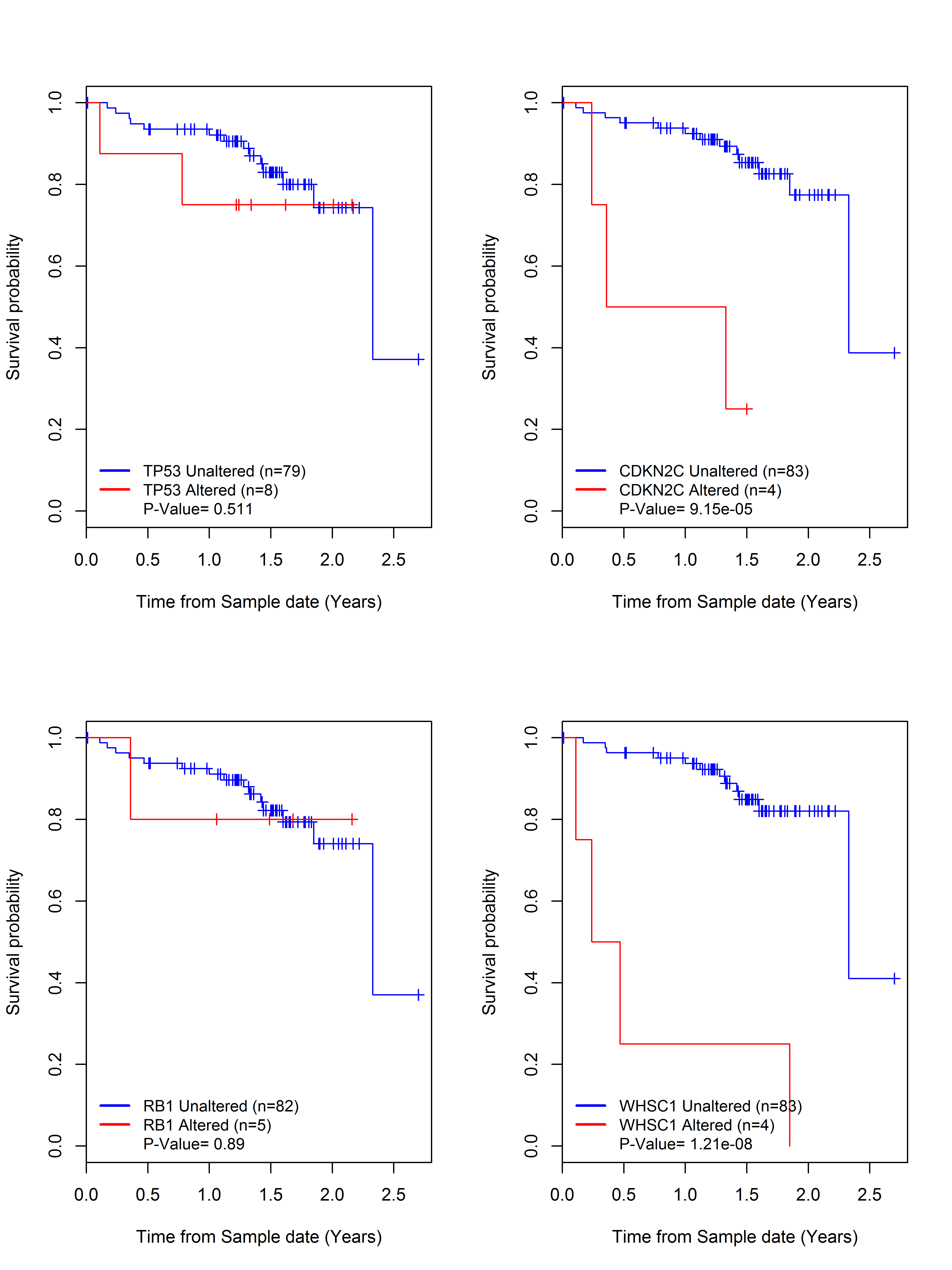


## Supplementary Figure 4. Kaplan-Meier plots for relapse myeloma (RLMM) for genes significant in multi-variate analysis.


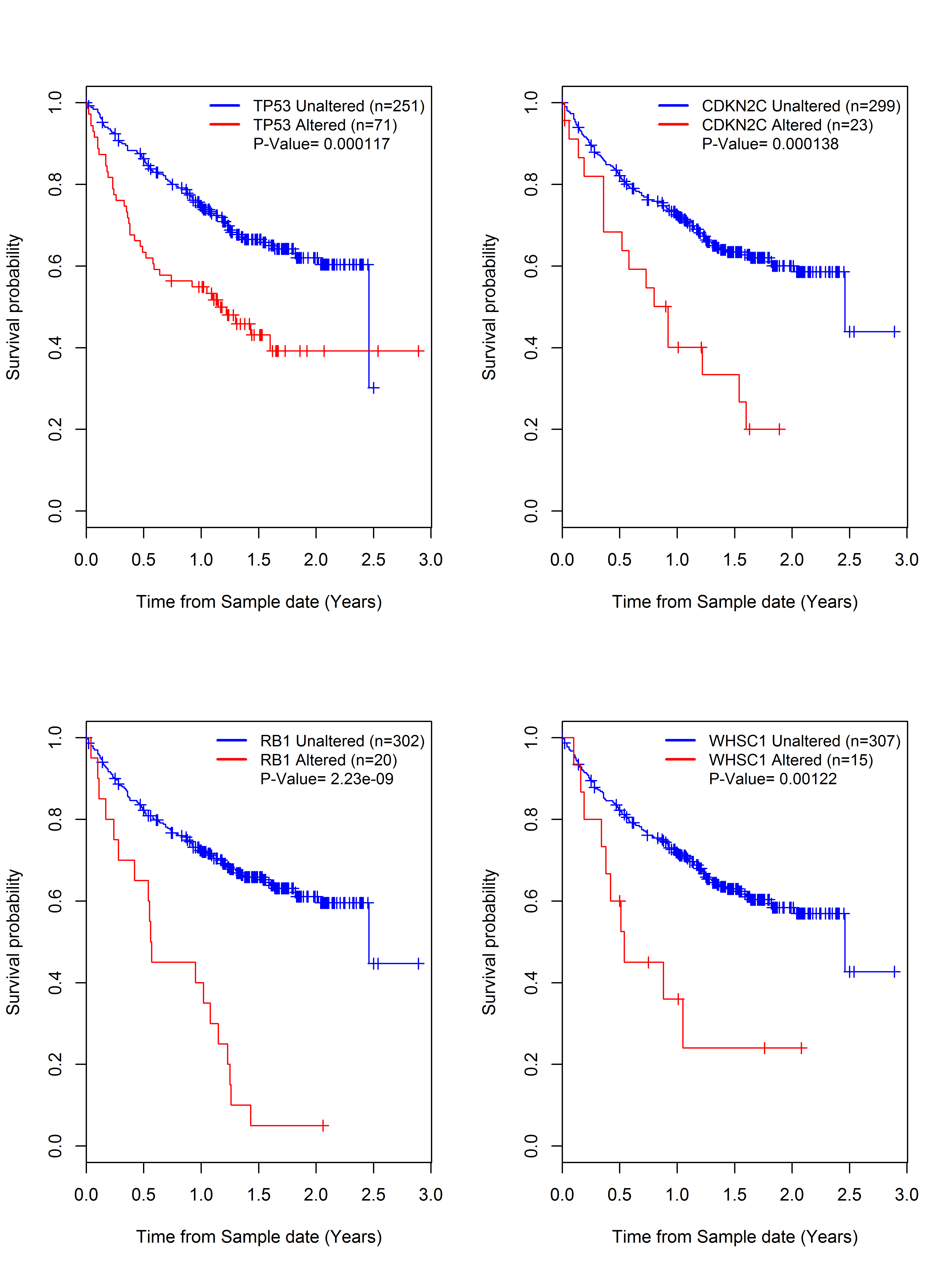


## Supplementary Figure 5. Kaplan-Meier plots for treated myeloma (TRMM) for genes significant in multi-variate analysis.


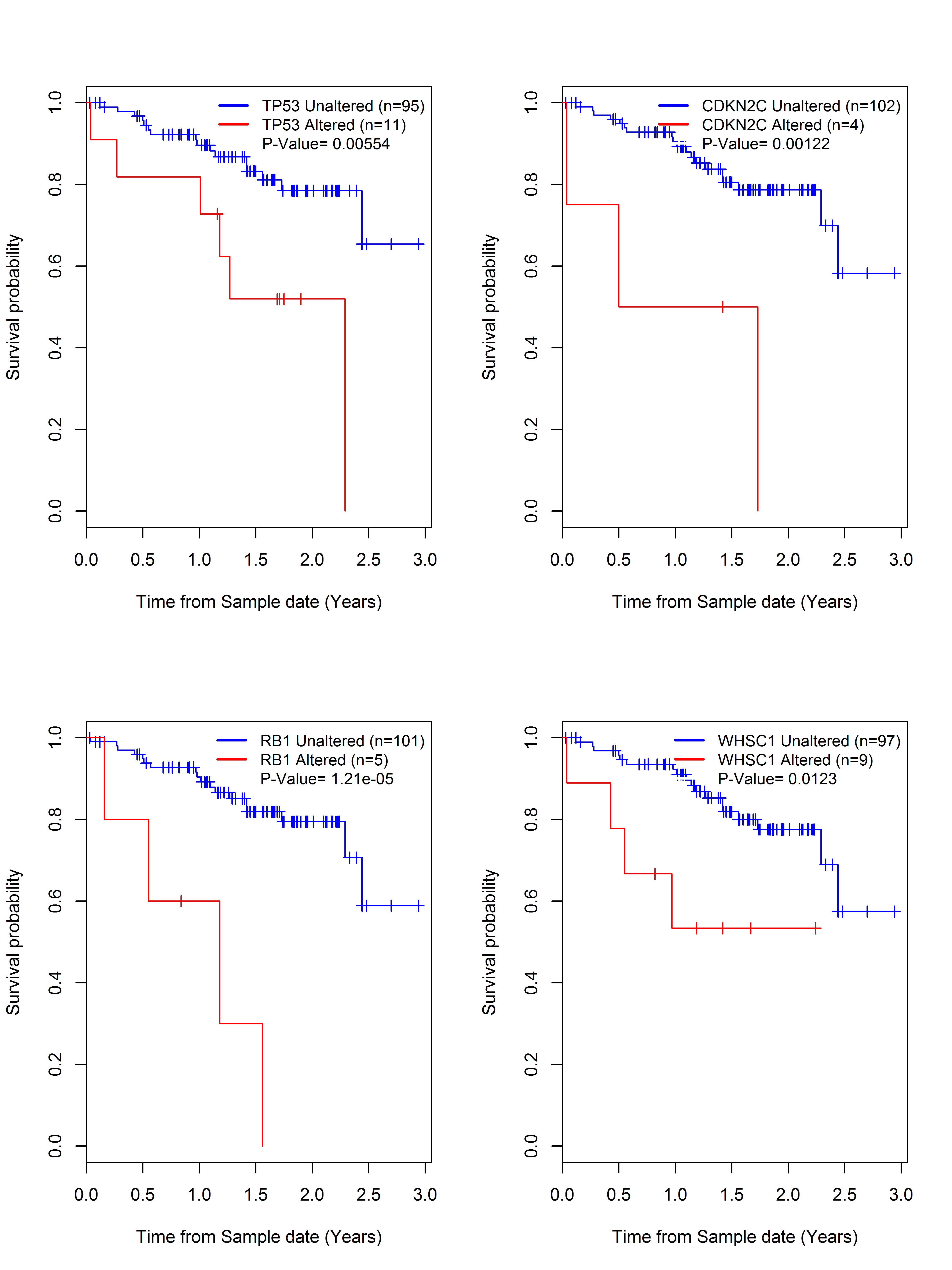


## Supplementary Figure 6. Comparison of frequency of alterations at different disease stages. A, MGUS vs. SMM; B, SMM vs. NDMM; C, NDMM vs. RLMM


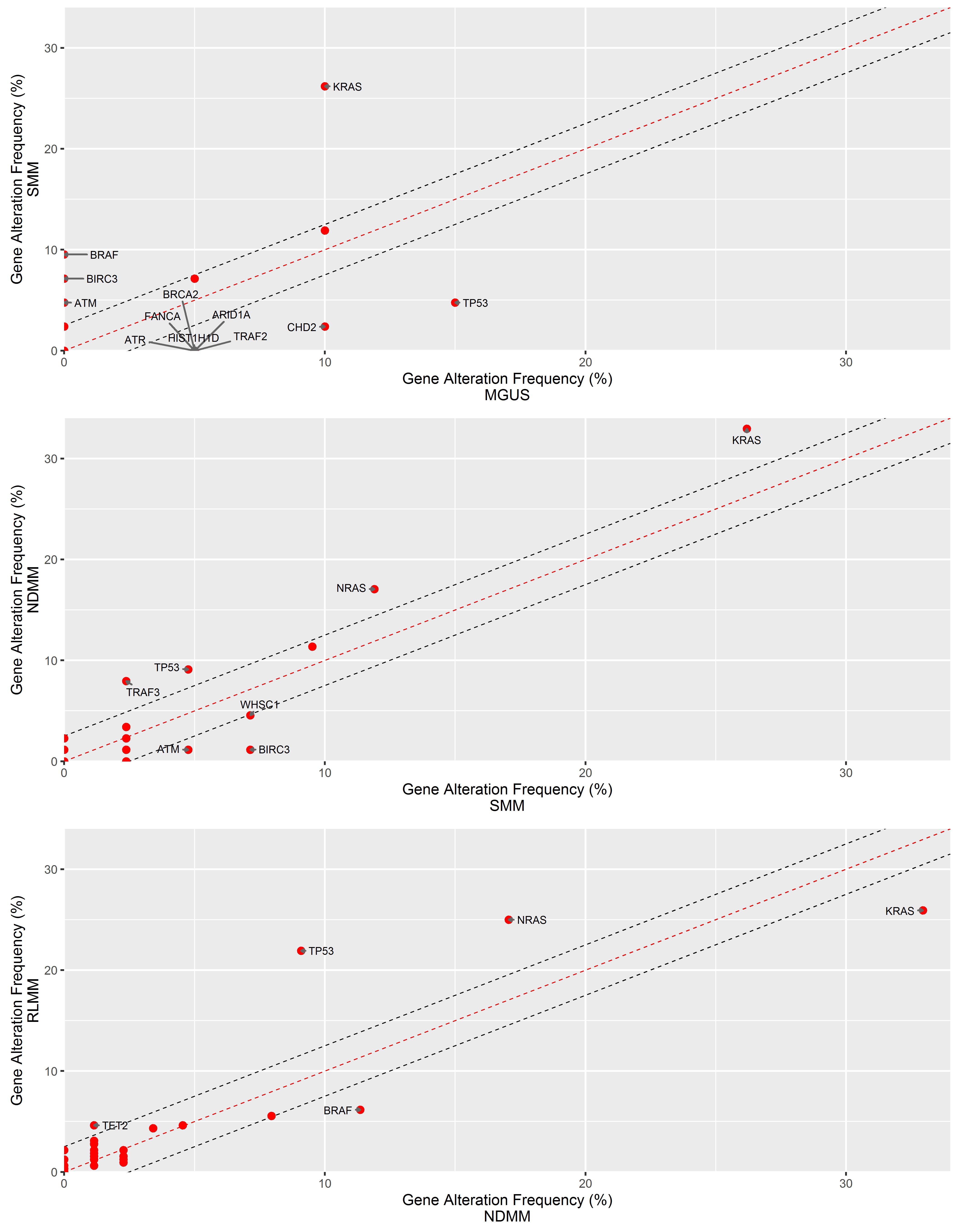


## Supplementary Figure 7. Comparison of allele frequency of gene mutations at different disease stages in KRAS, NRAS, BRAF, and TP53


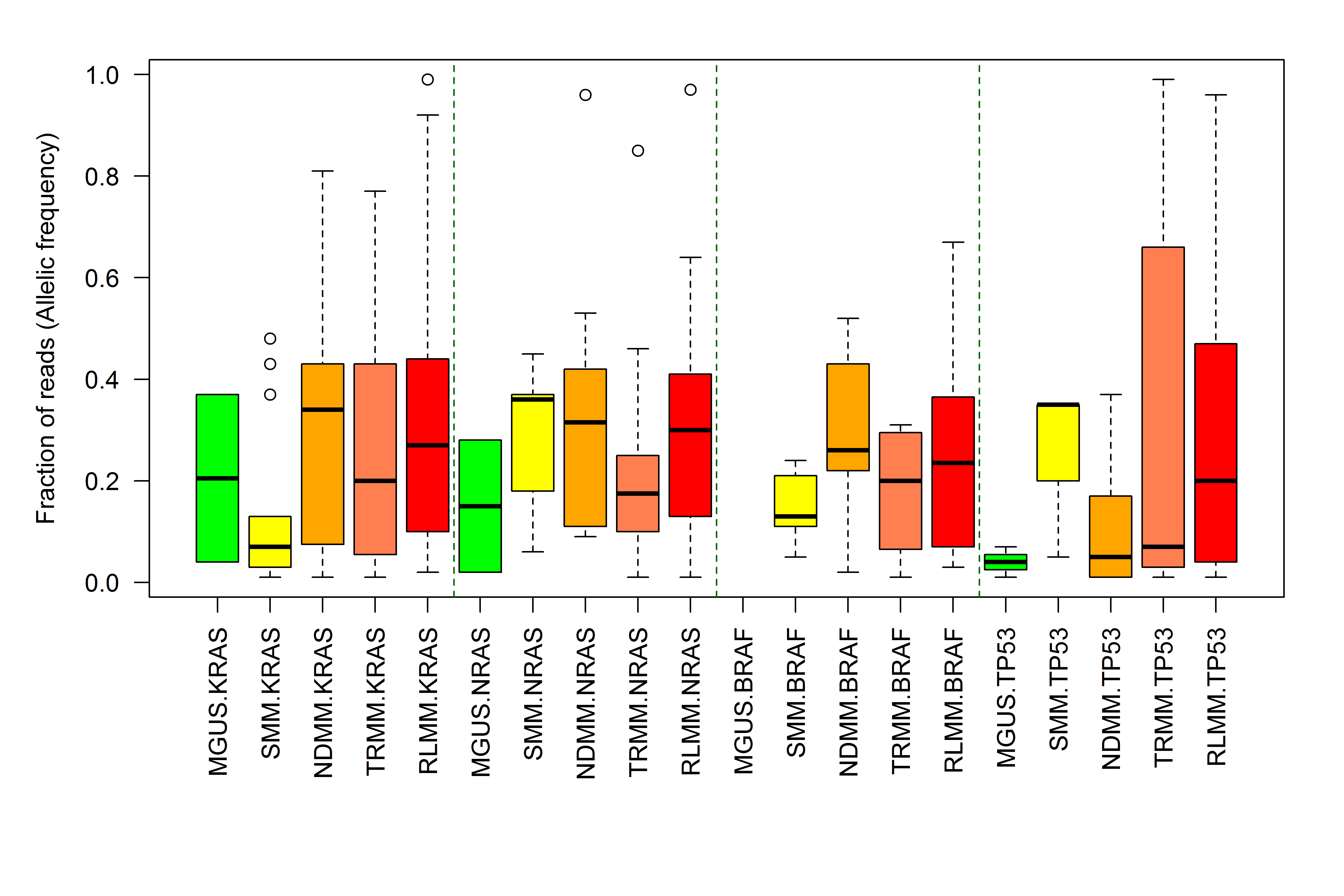


## Supplementary Figure 8. KRAS, but not NRAS or BRAF, alterations result in a worse overall survival.


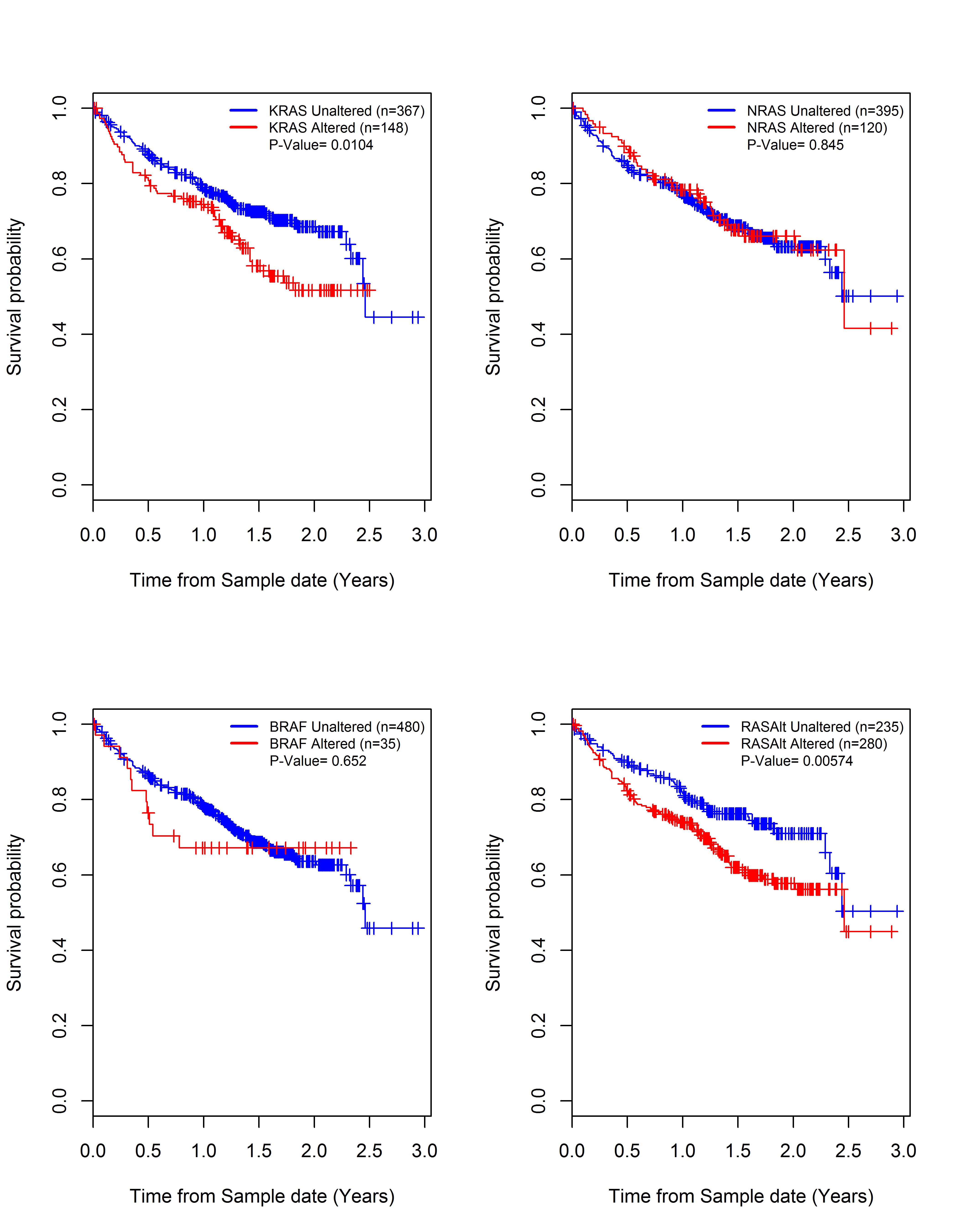


## Supplementary Figure 9. Effect of KRAS mutation at A. NDMM B.TRMM C.RLMM

**
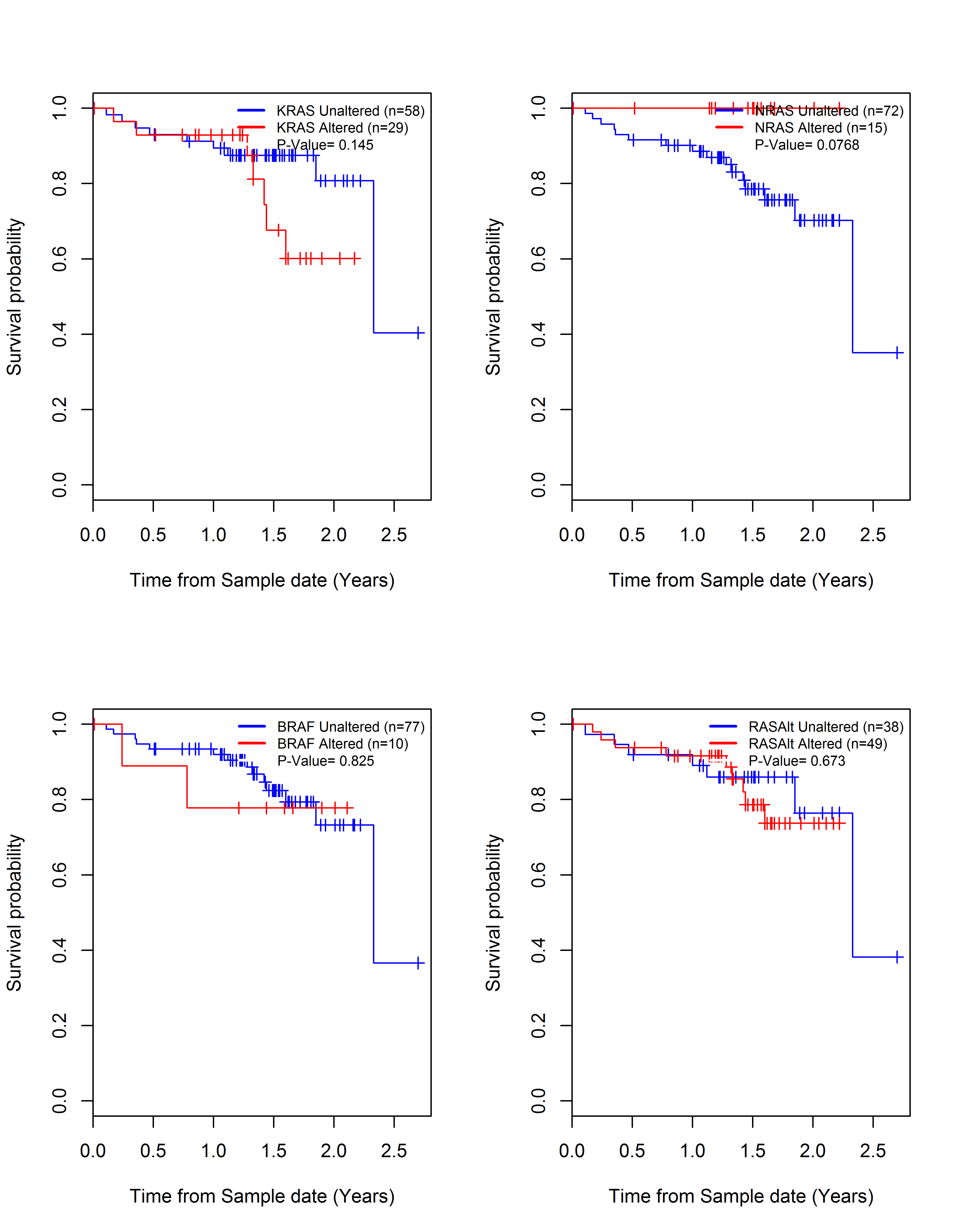

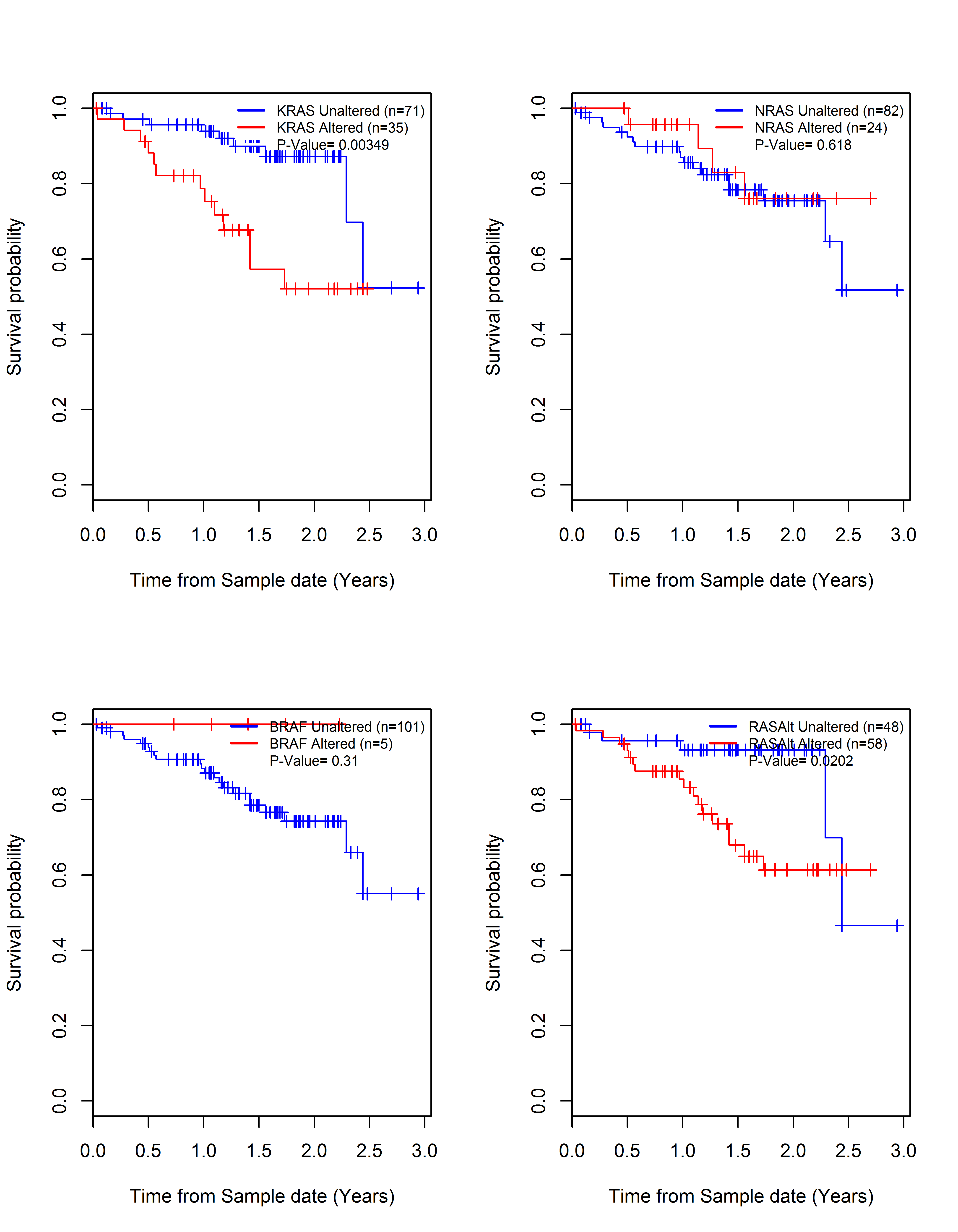

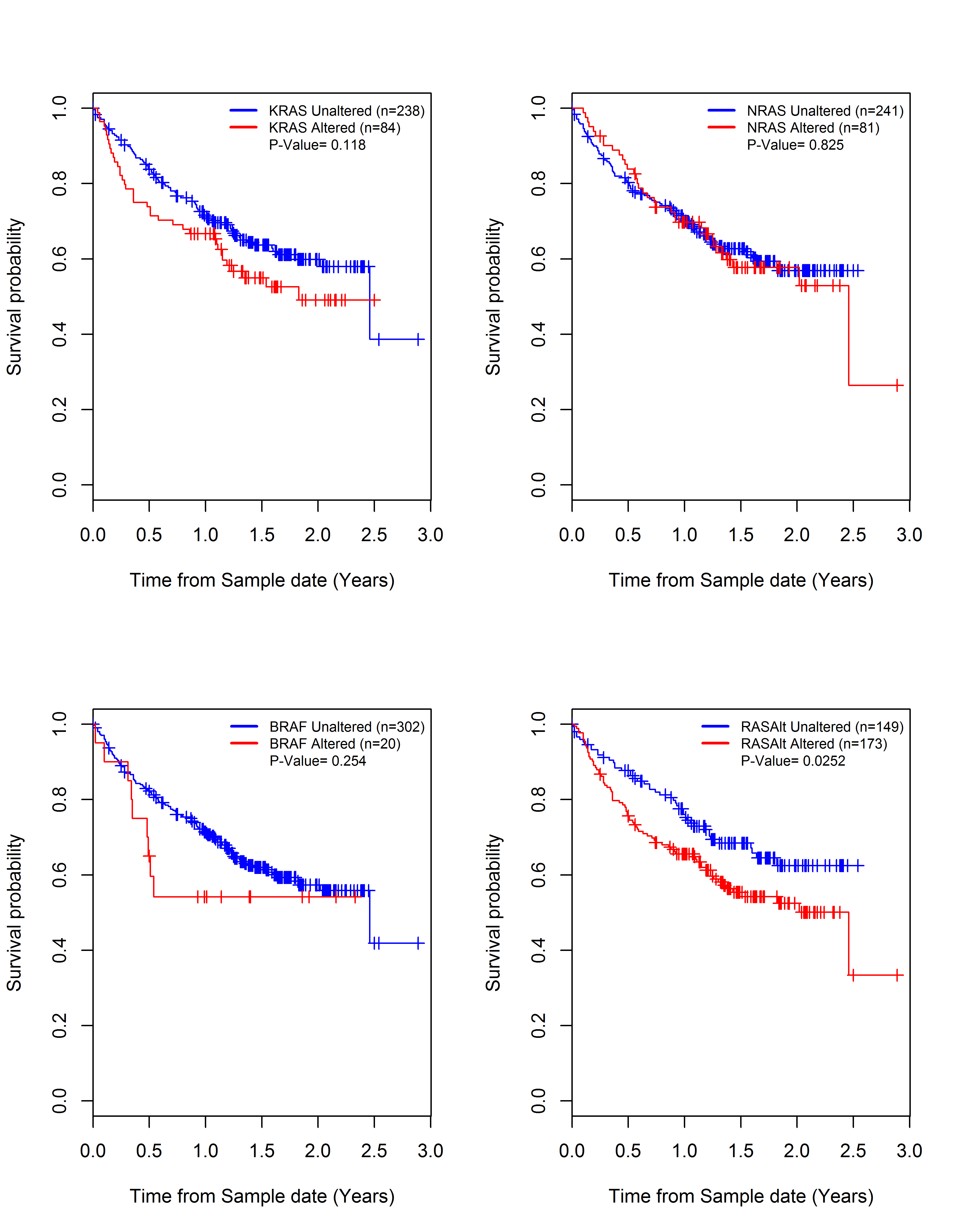
**
